# Evaluation of platelet parameters, coagulation markers, antiphospholipid syndrome, and thyroid function in palestinian women with recurrent pregnancy loss

**DOI:** 10.1186/s12884-023-05764-6

**Published:** 2023-06-20

**Authors:** Ayman A. Najjar, Imam Hassouna, Mahmoud A. Srour, Hany M. Ibrahim, Randa Y. Assi, Heba M. Abd El Latif

**Affiliations:** 1 Zoology Department, Faculty of Science, Menoufia University, Menoufia, Egypt; 2 Department of Biology and Biochemistry, Faculty of Science, Birzeit University, Birzeit, Palestine; 3 Clinical Laboratory Science program, Faculty of Pharmacy, Nursing and Health Professions, Birzeit University, Birzeit, Palestine; 4 Department of Obstetrics & Gynecology, Faculty of Medicine, Al-Quds University, Jerusalem, Palestine

**Keywords:** Antiphospholipid syndrome, Coagulation parameters, Thyroid function, Recurrent pregnancy loss, Platelet parameters, Palestine

## Abstract

**Background:**

Multiple etiologies contribute to recurrent pregnancy loss (RPL) including immunological, endocrine, anatomical, genetic and infection but more than 50% of cases remain unexplained. Evidences of thrombotic and inflammatory processes were observed at maternal-fetal interface and considered pathological findings in most RPL cases including unexplained cases. This study aimed to evaluate the association between RPL and several risk factors: platelet parameters, coagulation factors, antiphospholipid syndrome, and thyroid function.

**Methods:**

This is an unmatched case-control study that included 100 RPL and 100 control women. Anthropometric and health data were collected and a gynecologist examined participants to assure fitting the inclusion criteria. Platelet parameters [including Mean Platelet Mass (MPM), Concentration (MPC) and Volume (MPV)] and ratios (MPV/Platelet, MPC/Platelet, MPM/Platelet, Platelet/Mononuclear cells), coagulation markers [Protein C (PC), Protein S (PS), Antithrombin III, D-dimer], antiphospholipid antibodies [Anti-phospholipid (APA), Anti-cardiolipin (ACA) and anti-B2-glycoprotein 1], Lupus anticoagulant, Antinuclear antibodies, and thyroid function (Thyroid stimulating hormone and anti-thyroid peroxidase) were measured.

**Results:**

Mean ages of cases and controls at marriage were 22.5 years for both, and their current ages were 29.4 and 33.0, respectively. 92% of cases and 99% of controls aged blow 30 years at marriage. 75% of cases have 3–4 miscarriages and 9% have ≥ 7 miscarriages. Our results indicated significantly lower male/female age ratio (p = .019), PC (p = .036) and PS (p = .025) in cases compared to controls. Plasma D-dimer (p = .020) and antiphospholipid antibodies [ACA (IgM and IgG), APA (IgM)] were significantly higher in cases compared to controls. No significant differences were observed between cases and controls concerning APA (IgG), anti-B2-glycoprotein 1 (IgM and IgG), Lupus anticoagulant, Antinuclear antibodies, platelet parameters, thyroid markers, family history of miscarriage, consanguineous marriage, and other health data.

**Conclusions:**

This is the first study that investigated the association between platelet, coagulation, antiphospholipid, autoimmune and thyroid parameters, and RPL in Palestinian women. Significant associations between male/female age ratio, PC, PS, D-dimer, ACA (IgM, IgG), APA (IgM) and RPL were observed. These markers could be used in evaluating RPL. These findings confirm the heterogeneous nature of RPL and emphasize the need for further studies to find out risk factors for RPL.

**Supplementary Information:**

The online version contains supplementary material available at 10.1186/s12884-023-05764-6.

## Introduction

Around 2.5% of the women trying to conceive suffered from recurrent pregnancy loss [1]. About twenty-three million miscarriages happen every year worldwide, that means about forty-four pregnancy losses per minute and about 35 miscarriages occurred annually per 1000 women aged 14–44 years worldwide in the years 2010–2014 [2]. The incidence of infertility among married Palestinian women is 2.4% as primary infertility and 6.0% as secondary infertility [3]. The European Society for Human Reproductive and Embryology (ESHRE) and the American Society of Reproductive Medicine (ASRM) define recurrent pregnancy loss (RPL) as three or more consecutive pregnancy losses before 22 weeks of gestation [4–11]. RPL is also defined as the loss of a fetus at eighteenth gestational week if gestational age is unknown or the loss of a fetus that weighs < 400 g [5].

Pregnancy is a physiological challenge to the hemostatic system; it is a state of hypercoagulability and hypofibrinolysis to maintain placental function as a requirement for fetal survival, and to prevent excessive bleeding during delivery [12–14]. But these changes increase thrombogenicity and increase the risk of thrombosis 4 to 6-folds compared to non-pregnant women due to increased plasma levels of coagulation factors such as factors VII, VIII, X, vWF, fibrinogen and a decreased factor XI [15–19]. Most coagulation factors, proteins, enzymes and platelet parameters were altered during normal pregnancy, but still are balanced by hormonal and other alterations in plasma proteins [12–14]. Studies have shown that protein C (PC), protein S (PS), and anti-thrombin III (ATIII) are changed during the overall course of pregnancy to maintain fetal circulation [12]. Any changes in the unique pregnancy hemostasis may direct pregnancy toward the risk of thrombosis, bleeding or consequently miscarriage [12]. Worldwide, most studies about hemostasis and pregnancy have investigated blood coagulation factors, blood coagulation inhibitors, fibrinolytic markers, platelet function or anti-phospholipid antibodies (anti-cardiolipin (ACA), anti-phospholipid (APA) and anti-B2-glycoprotein 1 (B2GP1)), separately [14]. But it is more appropriate to study these factors and their interactions together.

Several risk factors contribute to RPL including uterine factors like anatomical defects and chronic endometritis, antiphospholipid syndrome, inherited thrombophilia, thyroid dysfunction, genetic factors, feto-maternal ABO incompatibility, environmental and psychological factors, as well as acquired factors like maternal age, medical conditions and medications [5, 6, 11, 20, 21]. However, the etiology of RPL is identified in less than half of RPL cases, and the mechanisms underlying it remain poorly understood, and thus present a challenge to the clinician [4, 21].

In the past 20 years, about five studies have investigated the correlation between inherited thrombophilia (IT) and RPL in Palestinian women from West Bank and Gaza [13, 22–25]. These reports focused on IT mutations only and up to our knowledge, other factors contributing to RPL such as antiphospholipid antibodies, coagulation and fibrinolytic activation markers, thyroid disorders and platelet parameters have not been investigated among Palestinian women. However, for establishing the etiology of RPL cases an array of laboratory tests implicated with RPL should be investigated including platelets parameters, coagulation markers (PC, PS, ATIII, D-dimer), antiphospholipid antibodies (APA, ACA, B2GP1, and Lupus anticoagulant) and thyroid function (TSH and anti-TPO). Thus, the current study aimed to evaluate the association between the aforementioned markers and RPL among Palestinian women.

## Materials and methods

### Study design and setting

This is an unmatched case-control study that included women with RPL and control women from West Bank region, Palestine. The Palestinian population in the West Bank region of Palestine was 2,986714 in June 2019 [3]. From the total population of the West Bank region, females accounted for 1,463489 (49%) and of whom 715,646 (48.9%) are in the childbearing age (15–49 years old). About 443,700 of reproductive age females are married. A non-probability sampling was used by setting a quota for each of the three regions of west bank (North, Middle, and South) relative to the population size of each region. After that we enrolled patients and controls at each region consecutively until the quota was fulfilled. A convenient sample size of 200 women (100 cases and 100 controls) was recruited from the different regions of the West Bank in the period from February 2020 to August 2020. Blood samples were collected from private medical centers representing the northern, central and southern regions of West Bank.

The inclusion criteria for study RPL group were at least three RPL before 22th weeks of gestation for unexplained reasons. A control group of 100 healthy women was recruited from the same areas of study group (RPL patients). Controls were selected to have no history of pregnancy loss and have at least two consecutive live births, to exclude secondary and tertiary RPL cases. Patients and controls stated that they were not using anticoagulant therapy (Enoxaparin sodium or aspirin) for at least one month before sample collection. Cases and controls that were on hormonal or anticoagulant therapy were excluded from the study. Additionally, RPL cases with three or more miscarriages and have at least one live birth were excluded. Both study groups were not pregnant at time of sample collection and absence of pregnancy was confirmed by quantitative B-hCG. All RPL patients stated that they did not use family planning contraceptive measures from time of marriage to the time of sample collection. A written informed consent was acquired from all subjects. General health and anthropometric data were collected from all study subjects using an interview-based questionnaire that was filled completely at the time of phlebotomy.

### Ethical approval

Ethical approval was obtained from the local ethics committee of the Palestinian Medical Technology Association (PMTA-EC meeting JAN2020 No.26/2020). Participants were briefed about the study and its objectives, given a written informed consent before being enrolled in the study. To ensure confidentiality, all information was rendered anonymous. All study protocols were performed in accordance with the Declaration of Helsinki.

### Sampling procedure

A short interview was conducted with all participants, asked to give a written informed consent and then were examined by a gynecologist who examined if the participant met the inclusion criteria. Then, an interview-based questionnaire was filled. Venous blood was collected by vacutainer tube method in three different tubes; Ethylene diamine tetra acetate (EDTA)- tube for complete blood count (CBC) and analysis of platelets parameters; citrated tube for analysis of PC, PS, LA, ATIII, and D-dimer; while serum (clot activator tube) was used for the analysis of TSH, Anti-TPO, ACA, APA, B2GP1 Abs, and ANA. All laboratory procedures were conducted based on the respective Standard Operating Procedures (SOPs). EDTA or citrated samples were gently mixed for 10 min on a special mixer to prevent clumping and clot formation, while plain tubes were incubated for 30 min to complete clotting. Plasma and serum were aliquoted into new labeled sterile tubes for analysis.

### Measurement of variables

CBC including platelet parameters was measured using a laser-based hematology analyzer ADVIA 2120i (Siemens Healthineers, Germany) that generates a higher quality measurement compared to impedance-based methodology. The platelet parameters measured include the routinely used parameters such as platelets count (PLTs), plateletcrit (PCT), mean platelet volume (MPV), platelet distribution width (PDW), platelet large cell ratio (PLCR) and platelet large cell concentration (PLCC); as well as the new platelet parameters; mean platelet mass (MPM) and mean platelet component concentration (MPC). These new platelets parameters are more informative than MPV and PDW as markers of platelet activity. ABO blood grouping and RhD antigen were phenotyped using standard laboratory techniques. PC, PS and LA were measured using Elisys Uno Fully Automated ELISA Analyzer (Human Company, Germany) following the manufacturer’s instructions and standard laboratory protocols. ATIII was measured by RID kit methodology from immunodiagnostic KENT laboratories (Bellingham, USA). TSH, anti-TPO and D-dimer were measured using Siemens IMMULITE 1000 Immunoassay analyzer (Siemens Healthineers). ACA, APA, B2GP1 and ANA antibodies were measured by ELISA kits according to the manual procedure of AskuLisa-Company (Germany) per the manufacturer’s instructions.

### Data quality assurance

Personal protective equipment was used throughout all steps. SOPs were followed while collecting the samples by a trained phlebotomist, samples were inspected for hemolysis, clotting, volume and collection time, and labeling after collection was done. The quality of samples and reagents was assured based on the respective SOP and the performance of the analyzers was maintained by running three levels of controls (Normal, Low, and High). Abnormal results were repeated twice to confirm the results.

### Data processing and analysis

Results were tabulated in excel sheet, and then the data were exported and analyzed using SPSS version 23. We insured having normal distribution by eliminating outliers without exceeding 5% of the data. Independent t-test was used to compare means of continuous variables and Chi-square for non-continuous variables. A *P*-value ≤ 0.05 was considered statistically significant.

## Results

### Characteristics of study population

A total of 200 study participants were recruited for participation in this study. The first group (RPL patients) included 100 women with unexplained RPL, and all have experienced three or more consecutive pregnancy losses before 22th weeks of gestation. The second group (controls) included 100 healthy women with at least two live children. The mean ages of RPL patients and controls at marriage was almost the same, but the mean current ages of RPL patients and controls were 29.4 and 33.0 years, respectively. Study subjects were recruited from West Bank regions of Palestine to yield a representative sample of the study region (West Bank) and to avoid geographical factors that may affect the measured parameters. The general characteristics of RPL patients and controls (Table 1) showed that all RPL patients and controls at marriage date are in the reproductive age and 92% of RPL patients and 99% of controls are less than 30 years old. The body mass index (BMI) for both RPL patients and controls showed that 53% of RPL patients and 43% of controls are in the normal BMI category while the rest was in the overweight categories, except for 2 underweight controls (Table 1). There were no significant differences between the average maternal ages at marriage for RPL patients versus controls. While the husband’s age at marriage was significantly lower in controls compared to RPL patients. Additionally, we calculated the male (husband’s age at marriage) to female (wife’s age at marriage) age ratio (M/F ratio; Table 1), in order to explore possible physiological effects related to age between husband and wife and to determine whether pregnancy outcome is influenced by couple’s age gap. RPL patients showed a significantly lower M/F ratio compared to controls (Table 1). The majority of RPL patients and controls have A and O blood groups and there were no significant differences between RPL patients and controls concerning Wife’s or husband’s blood groups (Table 1).

**Table 1:** General characteristics of study population (RPL patients and controls)

Characteristic	RPL Patients (n = 100)		Control (n = 100)		*P*- value
	Mean ± SD	Min-Max.	Mean ± SD	Min-Max.	
Wife’s age at marriage	22.5 ± 4.0	16–35	22.5 ± 3.0	17–33	0.920
Wife’s current age	29.4 ± 5.1	20–42	33.0 ± 5.3	21–45	0.011*
Husband’s age at marriage	27.2 ± 5.2	17–42	25.9 ± 3.7	19–36	0.047*
Husband’s current age	34.1 ± 6.2	23–50	36.5 ± 5.7	24–52	0.016*
M/F age ratio at marriage	1.2 ± 0.18	0.9–1.9	1.6 ± 0.34	1.1–3.1	0.019*
Wife’s age groups at marriage (n)					
• 16–19 years	25	--	17	--	0.166
• 20–29 years	67	--	82	--	0.015*
• 30–45 years	8	--	1	--	0.017*
Wife’s BMI, Kg/m^2^(M ± SD)	26.2 ± 4.8	16.4–42.4	25.8 ± 4.1	17.6–38.6	0.579
Wife’s BMI categories (n)					
• Underweight < 18.5	0	--	2	--	0.156
• Normal 18.5–24.9	53	--	43	--	0.158
• Overweight 25-29.9	27	--	40	--	0.052
• Obese ≥ 30	20	--	15	--	0.353
Wife’s blood groups (n)					
• A	38	--	33	--	0.461
• B	21	--	16	--	0.364
• AB	6	--	9	--	0.422
• O	35	--	42	--	0.310
• Rh(D) positive	90	--	94	--	0.298
• Rh(D) negative	10	--	6	--	0.298
Husband’s blood groups (n)					
• A	36	--	43	--	0.313
• B	7	--	14	--	0.107
• AB	18	--	10	--	0.104
• O	39	--	33	--	0.378
• Rh(D) positive	93	--	94	--	0.775
• Rh(D) negative	7	--	6	--	0.775

Data are expressed as mean ± SD for continuous variables, as well as the minimum and maximum values are presented. For nominal variable, data are expressed as numbers (n). The BMI was calculated as = weight in Kg/ (length in meters)^2^. SD: standard deviation. BMI: Body mass index. Statistical analysis was done using t-test for continuous variables and Chi-square for nominal variables

The study investigated ABO interactions, particularly ABO incompatibility cases that prevent the father from donating blood to the mother. Although blood types are different, A type mother and O type father will be ABO blood type compatible, but O type mother and A type father will be defined as ABO blood type incompatibility. Here we analyzed these combinations and examined whether ABO incompatibility could contribute to RPL. ABO-incompatible mating does not necessarily result in an ABO-incompatible fetus. The Wife’s/ Husband’s blood group combinations are shown in Table S1, and the incompatible combinations are shown in Table S2. The major incompatible combinations were O/A (Table S2). However, there were no significant differences between RPL patients and controls (Table S2).

Analysis of the family history of miscarriage, consanguineous marriage, and other health data of RPL patients and controls in Table 2 revealed that there is no significant difference between RPL patients and controls except for anticoagulant use and vitamin B12 use. Most RPL patients (75%) have 3–4 miscarriages and 9% have 7 or more miscarriages. Almost two-thirds of RPL patients were on anticoagulant therapy, while none of the controls used anticoagulants. The number of RPL patients using vitamin B12 supplements were significantly lower than the controls.

**Table 2:** Family history, history of chronic diseases, life style habits and use of vitamin supplements and anticoagulants of RPL patients and controls

Characteristic	RPL Patients (n = 100)	Controls (n = 100)	*P*-value
No. of miscarriages			
3–4	75	0	--
5–6	16	0	--
≥ 7	9	0	--
Family history of miscarriage	44	56	0.092
Consanguineous marriage	20	17	0.585
Diabetes mellitus	2	0	0.156
Hypertension	1	0	0.317
Family history of CVD	16	16	1.000
Anticoagulant use^1^	65	0	< 0.001*
Smoking	13	19	0.248
Coffee drinking	69	63	0.372
Vitamin B12 supplement^1^	27	54	< 0.001*
Folate supplement^1^	98	92	0.052

Data are expressed as number of subjects (n). Statistical analysis was done using Chi-square. CVD: Cardiovascular disease. ^1^These data represent the state of patients and controls during their last miscarriage or pregnancy, respectively

### Platelet parameters

The new platelet parameters (MPM and MPC) indicate platelet functions and activation better than the routinely used parameters (MPV and PDW).

Analysis of platelets count, indices and ratios showed no significant changes between RPL patients and controls (Table 3). Indeed, the means and medians of RPL patients and controls were very close for all platelet parameters analyzed in the current study.

**Table 3:** Comparison of the platelet’s parameters among RPL patients and controls

Parameter	RPL Patients (n = 100)				Controls (n = 100)				*P*-value
	Mean ± SD	Median	Min.	Max.	Mean ± SD	Median	Min.	Max.	
PLT count (K/uL)	262.3 ± 56.8	262	176	396	256.0 ± 73.4	256	130	430	0.501
MPV (fL)	8.4 ± 1.6	8.9	7.0	12.2	8.2 ± 1.3	9.2	7.0	12.4	0.325
PCT (%)	0.25 ± 0.05	0.24	0.14	0.34	0.23 ± 0.06	0.24	0.13	0.39	0.083
PDW (%)	53.2 ± 7.1	52.1	37.5	70.5	54.8 ± 8.3	54.7	34.5	69.9	0.135
MPC (g/dL)	23.7 ± 3.2	24.7	17.2	30.9	23.8 ± 2.6	24.4	17.9	28.8	0.707
MPM (pg)	2.06 ± 0.19	2.04	1.49	2.49	2.08 ± 0.21	2.08	1.60	2.40	0.420
PLCR (%)	2.9 ± 2.0	2.4	0	7.8	3.0 ± 2.0	2.6	0.2	8.3	0.929
PLCC (K/uL)	7.3 ± 4.2	6.0	0	17.0	7.3 ± 4.1	6.5	1.0	18	0.975
MPV/ PLT	3.7 ± 1.3	3.4	2.0	6.4	3.8 ± 1.4	3.5	2.0	7.8	0.650
MPC/ PLT	9.2 ± 2.0	8.9	5.3	14.6	9.7 ± 3.0	9.1	5.3	17.8	0.138
MPM/PLT	0.8 ± 0.2	0.75	0.4	1.3	0.8 ± 0.3	0.83	0.4	1.6	0.143
PLT/MN	8.4 ± 3.1	7.8	3.7	16.7	7.9 ± 2.6	7.7	3.5	14.2	0.242

Data are expressed as mean ± SD, median as well as the minimum and maximum values are presented. Statistical analysis was done using student t-test

### Coagulation parameters

Several coagulation parameters contribute to RPL. In this study, we have analyzed PC, PS, ATIII and D-dimer. As shown in Table 4, plasma levels of PC (103%) and PS (91%) in RPL cases were significantly lower than those recorded in controls (107%, 99%, respectively). However, there was no significant difference between RPL patients and controls concerning ATIII. The D-dimer level was significantly higher in RPL patients (223 ng/mL) compared to controls (184 ng/mL; Table 4).

**Table 4:** Comparison of RPL patients and controls based on the levels of PC, PS, D-dimer and ATIII.

	RPL Patients (n = 100)			Controls (n = 100)			*P-* value
	Mean ± SD	Median	Min - Max	Mean ± SD	Median	Min - Max	
PC %	103 ± 12	102	73–133	107 ± 10	107	80–128	0.036*
PS %	91 ± 8	91	70–111	99 ± 13	97	72–132	0.025*
D-dimer (ng/mL)	223 ± 144	194	50–1091	184 ± 72	178	69–497	0.020*
ATIII (mg/dL)	10.8 ± 0.9	10.7	9.2–15	11.2 ± 1.7	10.7	9.2–20.6	0.062

Data are expressed as mean ± SD, median as well as the minimum and maximum values are presented. Means were compared using student t-test. PC: protein C; PS: protein S; ATIII: antithrombin III

### Antiphospholipid syndrome

Antiphospholipid syndrome (APS) is a prothrombotic condition of autoantibody-induced thrombophilia. Analysis of the antibodies associated with APS in this study, revealed that plasma levels of ACA (IgM and IgG) and APA (IgM) in RPL patients were significantly higher than those found in controls. However, analysis of APA (IgG), B2GP1 (IgM and IgG), ANA and LA antibodies showed no significant differences between RPL patients and controls (Table 5).

**Table 5:** Comparison of RPL patients and controls based on presence of autoantibodies for APA, ACA, B2GP1, ANA and LA.

	RPL Patients (n = 100)			Controls (n = 100)			*P-* value
	Mean ± SD	Median	Min - Max	Mean ± SD	Median	Min - Max	
ACA IgM (U/mL)	2.7 ± 1.3	2.3	1.1–6.3	2.0 ± 0.7	1.8	1.1–3.7	0.029*
ACA IgG (U/mL)	2.9 ± 1.5	2.2	1.1–6.6	2.1 ± 0.9	1.8	1.1–5.2	0.025*
APA IgM (U/mL)	2.6 ± 1.4	2.1	1.0–6.2	2.0 ± 0.6	1.9	1.0–3.7	0.023*
APA IgG (U/mL)	2.0 ± 0.5	1.9	1.0–4.0	1.8 ± 0.7	1.7	1.0–3.9	0.162
B2GP1 IgM (U/mL)	2.4 ± 0.7	2.3	1.3–4.3	2.2 ± 0.8	2.1	1.1–4.2	0.078
B2GP1 IgG (U/mL)	2.3 ± 1.0	2.2	1.1–5.2	2.2 ± 0.6	2.0	1.5–4.6	0.141
ANA (index)	0.50 ± 0.20	0.5	0.24–1.1	0.54 ± 0.17	0.52	0.20–1.0	0.785
LA	Positive 4%			Positive 6%			0.518

Data are expressed as mean ± SD, median as well as the minimum and maximum values are presented for all variables except LA that is expressed as percentages (%). For continuous variables, means were compared using student t-test while for LA, the Chi-square was used. APA: antiphospholipid antibodies; ACA: anti- cardiolipin antibodies; B2GPI: Beta 2 glycoprotein 1 antibodies; ANA: anti-nuclear antibodies; LA: lupus anticoagulant

### Thyroid function

Normal thyroid function is known to be critical for reproduction and normal fetal and embryonic development. Analysis of plasma levels of TSH and Anti-TPO showed no significant difference between RPL patients and controls (Table 6).

**Table 6:** Comparison of RPL patients and controls based on TSH level and the presence of Anti-TPO.

	RPL Patients (n = 100)			Controls (n = 100)			*P* value
	Mean ± SD	Median	Min- Max	Mean ± SD	Median	Min- Max	
TSH (µIU/mL)	1.63 ± 0.85	1.42	0.01–3.88	1.88 ± 1.01	1.59	0.01 - 5.2	0.064
Anti-TPO (IU/mL)	11.8 ± 9.5	8.8	5.6 - 61.4	11.6 ± 3.6	10.6	6.8–23.2	0.805

Data are expressed as mean ± SD, median as well as the minimum and maximum values are presented. Means were compared using student t-test. TSH: thyroid stimulating hormone; Anti-TPO: anti-thyroid peroxidase antibodies

## Discussion

Lifestyle factors are known to affect sporadic miscarriage, but their contribution to RPL is still not known [4, 5, 26]. In this study, several lifestyle factors and anthropometric data were analyzed for the study population (100 RPL patients and 100 controls). Male to female age ratio (M/F) in RPL patients was significantly lower than that in controls. This result indicates that a higher male to female age ratio may be preferred. This finding may provide new strategy for future marriage decision [27] but still needs further studies to shed light on this point. Additionally, our results showed that there was no significant difference between the age of RPL patients and controls at time of marriage and there was a significant difference between both groups concerning the mean current age (Table 1). Most study participants are younger than 30 years of age and this includes 92% of RPL patients and 99% of controls (Table 1). In addition, both RPL patients (20–42 years) and controls (21–45 years) have similar age range. All RPL patients stated that they did not use family planning contraceptive measures, and all attempted to conceive directly after marriage. Thus, these findings indicate that age was not a risk factor for RPL, at least among our study population.

The BMI revealed no significant difference between RPL patients and controls (p = .579) and thus BMI is not associated with RPL among our study population. Our findings are consistent with previous reports [28]. In contrast a recent meta-analysis showed that women with underweight and BMI > 25 have 1.2-fold increased risk of RPL compared to those with normal BMI [11]. Additionally, there were 1.3-fold and 1.7-fold increased risk of further miscarriage in RPL population with BMI > 25 and BMI > 30, respectively [11]. Obesity has been shown to contribute to ovulatory problems and compromised ovarian response to ovulation inducing agents and gonadotropins and consequently leads to reduced success rates following assisted reproduction [29].

ABO incompatibility occurs in 20% of pregnancies, but only 20% of them develop hemolytic disease, which is less severe than Rh incompatibility and can lead to uterine miscarriage [30]. Blood group (BG) [31] and Rhesus factor (Rh) incompatibility may cause RPL due to antigen antibody interaction. The current study showed that blood group combinations associated with paternal ABO incompatibility are not significantly different between RPL patients and controls. Altogether, blood type evaluation of miscarried couples suggested that blood type incompatibility is not a risk factor for RPL. These findings are in contrary with most studies about ABO blood group incompatibility [30–33]. An explanation for this contradiction is that ABO incompatibility between couples (wife/ husband) does not necessarily lead to feto-maternal ABO incompatibility. Additionally, in case the fetus inherited paternal ABO type that is incompatible with maternal ABO type, then fetal red cells (carrying ABO antigens) gaining access to maternal circulation will be eliminated immediately by the maternal naturally occurring ABO antibodies (mostly IgM). Consequently, there will be no further consequences for feto-maternal ABO incompatibility. In addition, maternal ABO antibodies are mostly IgM and thus cannot cross the placenta and harm the fetus. Despite the presence of very low percentage of maternal ABO anti-A or anti-B antibodies of the IgG isotype, their effect on the fetus will be limited [34].

Analysis of family history of miscarriage, consanguineous marriage, diabetes mellitus, hypertension, family history of CVD, anticoagulant use (Enoxaparin sodium, aspirin) and coffee drinking revealed no significant differences between RPL patients and controls. Our findings concerning the association of consanguineous marriage and RPL are consistent with earlier studies [35, 36]. As for cigarette smoking, 13% of cases and 19% of controls were smokers. Smoking during pregnancy has been reported to be associated with low birthweight, placental abruption and sudden infant death syndrome [37]. In addition, the risk of miscarriage has been shown to increase by 1% per cigarette smoked per day [37]. Reports investigating the association of smoking with RPL were inconsistent and this may be explained by inaccuracy or inability to describe smoking behavior and quantity of smoked cigarettes. In consistency with our finding, a recent meta-analysis study reported that cigarette smoking does not increase the risk of RPL in the general population [11].

Vitamin B12 and folic acid catalyze the re-methylation of homocysteine to methionine that is essential for normal embryogenesis and embryonic growth [38, 39]. Therefore, deficiency of folate or/and vitamin B12 have been found in 20% of cases with neural tube defects (NTD) and RPL due to vascular dysfunction and impairment of normal embryogenesis. Additionally, the rate of spontaneous miscarriage has been shown to be increased significantly in women of fetuses with NTD [38]. In our study folate supplement is being used by the majority of RPL patients (98%) and controls (92%), as a lifestyle or prophylactic therapy by both groups when they decide to conceive. Therefore, this study revealed that there were no differences between cases and controls in using folate as a prophylactic supplement. Suetterlin et al., [38] showed that there was no significant difference in serum levels of B12 and folate in RPL cases compared to controls and thus the role of prophylactic supplementation with folate and B12 in preventing RPL is questionable. In contrast, another study reported controversial results [11] that taking folate supplements is associated with increased risk for RPL. However, in the later study [11] women taking folate supplements were older and required longer time to conceive compared to the control group. In our study, vitamin B12 supplement was used by cases (27%) and controls (54%). Folate is a common prophylactic therapy for women who plan to conceive in both groups while vitamin B12 is not. RPL cases focus on folate supplement as a requirement for conceiving rather than on vitamin B12 as a routine supplement. In contrast, controls use vitamin B12 as a common practice in Palestine.

Laser-based hematology analyzer ADVIA 2120i generates new platelet indices such as MPM and MPC, which are considered markers of platelet activation and function and over weights the classical platelet indices MPV and PDW. Nowadays MPM and MPC are used as predictors of many disorders such as thrombosis and may be used for prediction of RPL [40]. MPV as a marker of PLT reactivation was shown to increase in response to infection and inflammation while PLT count decreased in the same manner [41]. However, the ratio of MPV/PLT becomes clearer than MPV and PLTS separately [41]. MPM and MPC as PLT reactivation markers overcome the MPV; therefore, this study used the ratios of MPM/PLT and MPC/PLT alongside the MPV/PLT.

Our results indicated that there was no significant association between platelets count, indices and ratios with RPL among Palestinian women. Thus, the investigation of platelet parameters is not useful for predicting RPL and thus other etiological factors should be sought. Previous studies reported that platelet parameters (PLT, PCT, MPV and PDW) are not associated with RPL [6, 32, 42]. A study conducted on 60 healthy controls and 45 hypertension patients and using ADVIA 120 hematology analyzer/system proved the usefulness of platelet parameters PDW and MPV as predictor for platelets activation [43].

Reduced levels of PC, PS, and ATIII and an elevated level of D-dimer indicate an activation of the coagulation process. In this study, we found a significant decrease in PC and PS levels in RPL patients compared to controls, but the reduced level of ATIII levels in RPL patients was not significantly different from that in controls. These findings are compatible with earlier reports that recommended testing for PC, PS, and ATIII in cases of RPL with unexplained etiology [10, 44]. However, a recent study found that low ATIII level is associated with RPL and recommended this test for the screening of unexplained RPL cases [45]. Additionally, we found significantly elevated levels of D-dimer in RPL patients compared to controls, which is consistent with earlier studies that found women with miscarriages have increased D-Dimer level when compared to normal early pregnancy [45–47]. Previous reports showed conflicting results concerning the D-dimer levels in cases of RPL and due to the large fluctuation in D-dimer results during the three trimesters of normal pregnancy [10, 48]. Thus, and based on our findings, investigation of physiological coagulation inhibitors (PC, PS, ATIII) and D-dimer should contribute to the explanation of the hypercoagulable state in RPL.

The maternal immune system does not ignore the fetus, but first recognizes and then responds to the employee antigens during development, and the physiological maternal tolerance towards the embryo ensures a successful pregnancy [8]. Antiphospholipid syndrome (APS) is a prothrombotic condition of autoantibody-induced thrombophilia [49]. APS autoantibodies include APA, ACA, B2GP1 and LA [20, 49, 50]. All these autoimmune antibodies target antiphospholipid-bound protein and may affect pregnancy and cause RPL [17, 49, 50]. APS can influence any body organ, which increases the risk for thrombosis, myocardial infarction, accelerated atherosclerosis, and stroke [49].

In this study, we found an association between APS autoantibodies level and RPL and in case of ACA (IgM, IgG) and APA (IgM). The level of the later antibodies was significantly higher in RPL patients compared to controls. Our findings are compatible with a previous study reporting that APA (IgM) are more accurate and have higher sensitivity than other antibodies [9, 50–52]. Although the level of APA (IgG), B2GP1 (IgM, IgG) was higher in RPL patients compared to controls, this difference was not statistically significant, thus indicating the absence of association between these antibodies and RPL at least among our study population. APS is a major reason for fetal loss due to a thrombotic tendency resulting in placental infarction during pregnancy [18, 50–52]. The prevalence of APS antibodies in healthy blood donors was recorded as 6.5% and 9.4% for ACA antibodies IgG and IgM, respectively [49].

Acquired autoantibody, LA, binds to phospholipids’ active coagulation factors and its action is not fully understood [17]. A previous study recorded an association of 7–10% between LA and recurrent spontaneous miscarriages in the 1st trimester [17]. LA is also detectable in viral, bacterial and parasitic infections [17]. In addition, LA is affected by many drugs [17]. Thus, these points must be taken into consideration when evaluating the LA. In this study, there was no association between LA and ANA on one side and RPL on the other side among our study population. Our findings confirm a previous report [17]. As for the ANA, there is no agreement on the clinical significance of it in association of RPL and it is considered as ancillary test in case of RPL [53]. However, a study found that ANA is not elevated in women with RPL [54]. Very recently, it has been demonstrated that the exact mechanism of ANAs in women with RPL is not clear, suggesting the correlation between ANAs and RPL and indicating its prognostic value for the subsequent pregnancy outcome [55].

An explanation for the variation in the prevalence of APS autoantibodies among different reports may be due to the average age of study cases as APS autoantibodies usually develop in older ages. Other explanations may include the different testing systems and the cut-off of each testing system. The genetic background of the different study cohorts may also play a role in the variations among the findings of different studies.

To investigate whether thyroid dysfunction could be associated with RPL we measured TSH and anti-TPO. Our results revealed that there was no significant difference between the means of TSH and anti-TPO in RPL patients and controls. This finding is compatible with a recent systemic review and meta-analysis that found no association between RPL and subclinical hypothyroidism and did not support screening for thyroid antibody in cases of RPL [7, 26, 56]. A cohort study of four hundred ninety-six women suffering from recurrent miscarriage of unknown etiology and two hundred and twenty women with a diagnosed etiology of recurrent miscarriage revealed that thyroid antibodies had no prognostic value concerning the outcome of future pregnancies in women with any reason of recurrent miscarriage [4].

Some limitations of this study is that only one blood sample was collected from RPL patients, although the study of hypercoagulopathy kinetics at different time points starting prior to pregnancy, during pregnancy and at time of miscarriage could provide a better insight about the role of hypercoagulopathy in the miscarriage process. Platelet aggregation studies could not be performed, although this marker may tackle further aspects of the role of platelets in the miscarriage alongside the extended platelets’ panel analyzed in this study including the new platelet activation parameters (MPM and MPC). Additionally, the fetus ABO blood group was not tested because of the lack of blood samples from miscarried fetuses. Feto-maternal ABO blood group incompatibility could provide a better view of the role of ABO incompatibility in miscarriage compared to the ABO blood group incompatibility between the father and the mother. Additionally, the sample size is small and a larger sample size shall enhance the generalization of the study findings.

## Conclusions

This study evaluated the association between different risk factors and RPL among Palestinian women. Our findings indicated that there were significant associations between M/F age ratio, PC, PS, D-dimer, ACA (IgM, IgG), APA (IgM) and RPL. Thus, these markers could be used in the evaluation of RPL. Other markers investigated in this study including a panel of platelet markers (such as the new MPC and MPM parameters) did show an association with RPL. The findings of this study confirm the heterogeneous nature of RPL and emphasize the need for further studies to find out risk factors for RPL.

Future studies that investigate the hypercoagulopathy kinetics at different time points throughout the pregnancy period, as well as platelets aggregation studies and fetal and paternal ABO phenotypes will contribute to a better understanding of the role of these factors in RPL.

## Electronic supplementary material

Below is the link to the electronic supplementary material.

Supplementary Material 1

## Data Availability

The datasets analyzed during the current study are available from the corresponding author on reasonable request and institutional authorization.

## List of Abbreviations

RPL: Recurrent pregnancy loss
PC: Protein C
PS: Protein S
ATIII: Antithrombin III
APA: Anti-phospholipid antibody
ACA: Anti-cardiolipin antibody
B2GP1: Anti-B2-glycoprotein 1
ANA: Anti-nuclear antibody
LA: Lupus anticoagulant
TSH: Thyroid stimulating hormone
anti-TPO: Anti-thyroid peroxidase antibody
M/F ratio: Male/female ratio
PLT: Platelets
MPV: Mean platelet volume
PCT: Platelet-crit
PDW: Platelets distribution width
MPC: Mean platelets components
MPM: Mean platelets mass
PLCR: Platelets large cell Ratio
PLCC: Platelets large cell concentration
MN: Mononuclear

## References

[1] Dimitriadis E, Menkhorst E, Saito S, Kutteh WH, Brosens JJ (2020). Recurrent pregnancy loss. Nat Rev Dis Primers.

[2] Sedgh G, Bearak J, Singh S, Bankole A, Popinchalk A, Ganatra B (2016). Abortion incidence between 1990 and 2014: global, regional, and subregional levels and trends. Lancet.

[3] Palestinian national bureau of statistics, Palestine, Population I. 2022. https://www.pcbs.gov.ps/site/lang__en/881/default.aspx#Population

[4] Morley LC, Shillito J, Tang T (2013). Preventing recurrent miscarriage of unknown aetiology. Obstetrician & Gynaecol.

[5] El Hachem H, Crepaux V, May-Panloup P, Descamps P, Legendre G, Bouet PE (2017). Recurrent pregnancy loss: current perspectives. Int J Women’s Health.

[6] Avcıoğlu SN, Altınkaya S, Küçük M, Sezer SD, Yüksel H (2016). The association between platelet indices and clinical parameters in recurrent pregnancy loss. Gynecol Obstet Reprod Med.

[7] Dong AC, Morgan J, Kane M, Stagnaro-Green A, Stephenson MD (2020). Subclinical hypothyroidism and thyroid autoimmunity in recurrent pregnancy loss: a systematic review and meta-analysis. Fertil Steril.

[8] Vomstein K, Feil K, Strobel L, Aulitzky A, Hofer-Tollinger S, Kuon RJ (2021). Immunological risk factors in recurrent pregnancy loss: Guidelines Versus Current State of the art. J Clin Med.

[9] Jabber YM, Hassan AJ, Abdullah HN (2021). The relationship between some immunological criteria and women with recurrent miscarriages. Ann Romanian Soc Cell Biol.

[10] Mekaj Y, Lulaj S, Daci F, Rafuna N, Miftari E, Hoxha H (2015). Prevalence and role of antithrombin III, protein C and protein S deficiencies and activated protein C resistance in Kosovo women with recurrent pregnancy loss during the first trimester of pregnancy. J Hum Reprod Sci.

[11] Ng K, Cherian G, Kermack AJ, Bailey S, Macklon N, Sunkara SK (2021). Systematic review and meta-analysis of female lifestyle factors and risk of recurrent pregnancy loss. Sci Rep.

[12] Moiz B. A review of Hemostasis in normal pregnancy and puerperium. Nat J Health Sci. 2017;3123–7. 10.21089/njhs.23.0123

[13] Ashour MJ, Sharif FA (2015). The relationship between gene polymorphisms of coagulation factors II, V and XI and risk of recurrent pregnancy loss in Palestine. Int Res J Med Med Sci.

[14] Hellgren M (2003). Hemostasis during normal pregnancy and puerperium. Sem Thromb Hemost.

[15] Battinelli EM, Marshall A, Connors JM. The role of thrombophilia in pregnancy. Thrombosis. 2013;516420. 10.1155/2013/516420 10.1155/2013/516420 PMC3880751 24455235

[16] Hong Li Y, Marren A (2018). Recurrent pregnancy loss: a summary of international evidence-based guidelines and practice. Aust J Gen Pract.

[17] Olaniyi J, Olomu S, Finomo O (2011). Lupus anticoagulant (LA) in pregnant women with history of recurrent fetal loss. J Blood Med.

[18] Suzumori N, Obayashi S, Kumagai K, Goto S, Yoshida A, Sugiura-Ogasawara M. A case of Microangiopathic Antiphospholipid-Associated Syndromes during pregnancy: review of the literature. Case Rep Med. 2012;827543. 10.1155/2012/827543 10.1155/2012/827543 PMC3395268 22811728

[19] D’Uva M, Micco PD, Strina I, Placido GD (2010). Recurrent pregnancy loss and thrombophilia. J Clin Med Res.

[20] Practice Committee of the American Society for Reproductive Medicine (2012). Evaluation and treatment of recurrent pregnancy loss: a committee opinion. Fertil Steril.

[21] Magnus MC, Wilcox AJ, Morken NH, Weinberg CR, Håberg SE (2019). Role of maternal age and pregnancy history in risk of miscarriage: prospective register based study. BMJ.

[22] Al-Astal MG, Sharif FA (2014). Beta-fibrinogen (-455 G/A) and integrin beta-3 (PLA1/A2) polymorphisms and recurrent pregnancy loss in Gaza strip-Palestine. Int J Reprod Contracept Obstet Gynecol.

[23] Abu-Asab NS, Ayesh SK, Ateeq RO, Nassar SM, El-Sharif WA. Association of inherited thrombophilia with recurrent pregnancy loss in palestinian women. Obstet Gynecol Int. 2011;689684. 10.1155/2011/689684 10.1155/2011/689684 PMC3135069 21765836

[24] Hussein AS, Darwish H, Shelbayeh K (2010). Association between factor V Leiden mutation and poor pregnancy outcomes among palestinian women. Thromb Res.

[25] Attili R, Hussein A, Odeh H, Hejaz H (2019). Prevalence of Thrombophilia in Palestine and the Association of Thrombophilic Gene polymorphisms with recurrent pregnancy loss. Res J Obstet Gynecol.

[26] Toth B, Würfel W, Bohlmann M (2018). Recurrent miscarriage: diagnostic and therapeutic procedures. Guideline of the DGGG, OEGGG and SGGG (S2k-Level, AWMF Registry Number 015/050). Geburtshilfe Frauenheilkd.

[27] du Fossé N, van der Hoorn ML, Eikmans M, Heidt S, le Cessie S, Mulders A (2019). Evaluating the role of paternal factors in aetiology and prognosis of recurrent pregnancy loss: study protocol for a hospital-based multicentre case-control study and cohort study (REMI III project). BMJ open.

[28] Kiremitli T, Kiremitli S, Ulug P, Dinc K, Uzel K, Arslan YK (2021). Are the body shape index, the body roundness index and waist-to-hip ratio better than BMI to predict recurrent pregnancy loss?. Reprod Med Biol.

[29] Pandey S, Maheshwari A, Bhattacharya S. Should access to fertility treatment be determined by female body mass index? Hum Reprod. 2010;25(4):815 – 20. 10.1093/humrep/deq013. Epub 2010 Feb 3. PMID: 20129994. 10.1093/humrep/deq013 20129994

[30] Hassanzadeh-Nazarabadi M, Shekouhi S, Seif N (2012). The incidence of spontaneous abortion in mothers with Blood Group O compared with other blood types. Int J Mol Cell Med.

[31] Ghasemi N, Sheikhha M, Davar R, Soleimanian S (2011). ABO blood groups incompatibility in recurrent abortion. Iran J Pediat Hematol Oncol.

[32] Akdemir N, Cevrioglu A, Ozden S, Kuru B, Bilir F, Bilir C (2013). Platelet indices and blood groups in early recurrent miscarriage: a study in pregnant women. J Clin Gynecol Obstet.

[33] Al-Fartosi KGW (2008). The association between ABO blood group and spontaneous abortion. Basrah J Sci.

[34] Akanmu AS, Oyedeji OA, Adeyemo TA, Ogbenna AA. Estimating the risk of ABO hemolytic disease of the Newborn in Lagos. J Blood Transfus. 2015;560738. 10.1155/2015/560738 10.1155/2015/560738 PMC4600530 26491605

[35] Najafi K, Mehrjoo Z, Ardalani F, Ghaderi-Sohi S, Kariminejad A, Kariminejad R (2021). Identifying the causes of recurrent pregnancy loss in consanguineous couples using the products of miscarriage with no chromosomal abnormalities. Sci Rep.

[36] Saad FA, Jauniaux E (2002). Recurrent early pregnancy loss and consanguinity. Reprod Biomed Online.

[37] Pineles BL, Park E, Samet JM (2014). Systematic review and meta-analysis of miscarriage and maternal exposure to tobacco smoke during pregnancy. Am J Epidemiol.

[38] Sütterlin M, Bussen S, Ruppert D, Steck T. Serum levels of folate and cobalamin in women with recurrent spontaneous abortion. Hum Reprod. 1997;12(10):2292-6. 10.1093/humrep/12.10.2292. PMID: 9402298. 10.1093/humrep/12.10.2292 9402298

[39] Reznikoff-Etiévant MF, Zittoun J, Vaylet C, Pernet P, Milliez J. Low Vitamin B(12) level as a risk factor for very early recurrent abortion. Eur J Obstet Gynecol Reprod Biol. 2002;104(2):156-9. 10.1016/s0301-2115(02)00100-8. PMID: 12206930. 10.1016/s0301-2115(02)00100-8 12206930

[40] Siemens. Advia 2021/2021i hematology system, # 067 D0157-01 rev. Siemens. 2010. https://www.manualscat.com/en/advia-2120i-manual

[41] Oh GH, Chung SP, Park YS, Hong JH, Lee HS, Chung HS et al. Mean platelet volume to platelet count ratio as a promising predictor of early mortality in severe Sepsis. Shock (Augusta, Ga.). 2017;47(3):323–30. 10.1097/SHK.0000000000000718 10.1097/SHK.0000000000000718 27504801

[42] Desouky R, Makled A, Abuelghar W, Mostafa R (2021). The association between platelet indices and unexplained recurrent miscarriage. Evidence-Based Women’s Health J.

[43] Boos CJ, Beevers GD, Lip GY (2007). Assessment of platelet activation indices using the ADVIATM 120 amongst ‘high-risk’ patients with hypertension. Ann Med.

[44] Mukhtar B, Garg R, Ibrahim G, Batra J (2023). Investigating protein C and S levels in pregnant women with recurrent early pregnancy loss versus normal pregnancy. J Med Life.

[45] Ye M, Fang R, Lun S, Fang J (2022). The association of AT-III and D-Dimer with unexplained recurrent spontaneous abortion and their diagnostic value for prethrombotic state. Am J Transl Res.

[46] Eichinger S (2004). D-dimer testing in pregnancy. Pathophysiol Haemost Thromb.

[47] Zeng J, Li Y, Dong Y, Chen Y, Liu Y, Wang S (2020). Predictive values of D-dimer for adverse pregnancy outcomes: a retrospective study. Clin Chem Lab Med.

[48] Hedengran KK, Andersen MR, Stender S, Szecsi PB. Large D-Dimer fluctuation in normal pregnancy: a longitudinal cohort study of 4,117 samples from 714 healthy danish women. Obstet Gynecol Int. 2016;3561675. 10.1155/2016/3561675 10.1155/2016/3561675 PMC4852125 27190521

[49] Corban MT, Duarte-Garcia A, McBane RD, Matteson EL, Lerman LO, Lerman A (2017). Antiphospholipid syndrome: role of vascular endothelial cells and implications for risk stratification and targeted therapeutics. J Am Coll Cardiol.

[50] Yang R, Zhang J, Zhang L, Liu Y, Guo Q (2022). Combined detection of anticardiolipin and anti-β2-glycoprotein 1 antibodies may predict pregnancy outcome. Am J Transl Res.

[51] Shaikhomar OA, Ali ST. A Comparative Analysis of Anticardiolipin, Anti-Β2-Glycoprotein-1, and Lupus Anticoagulants in Saudi Women with Recurrent Spontaneous Abortions. J Pers Med. 2022;13(1):2. Published 2022 Dec 20. 10.3390/jpm13010002 10.3390/jpm13010002 PMC9861935 36675663

[52] Hamadi GM, Lafta SF (2022). Immunological parameters of recurrent miscarriages among women in Thi-Qar province. J Med Life.

[53] Ticconi C, Rotondi F, Veglia M, Pietropolli A, Bernardini S, Ria F (2010). Antinuclear autoantibodies in women with recurrent pregnancy loss. Am J Reprod Immunol (New York N Y : 1989).

[54] Hefler-Frischmuth KHF, Walch K, Hefler L, Tempfer C, Grimm C. Serologic markers of autoimmunity in women with recurrent pregnancy loss. Am J Reprod Immunol. 2017;7(4). 10.1111/aji.12635. 10.1111/aji.12635. (New York, N.Y.: 1989). 28132421

[55] Liu T, Guo X, Liao Y, Liu Y, Zhu Y, Chen X (2022). Correlation between the presence of antinuclear antibodies and recurrent pregnancy loss: a Mini Review. Front Endocrinol.

[56] van Dijk MM, Vissenberg R, Bisschop PH, Dawood F, van Wely M, Goddijn M (2016). Is subclinical hypothyroidism associated with lower live birth rates in women who have experienced unexplained recurrent miscarriage?. Reprod Biomed Online.

