# Supplementary material for: Evaluation of platelet parameters, coagulation markers, antiphospholipid syndrome, and thyroid function in palestinian women with recurrent pregnancy loss
Source: BMC Pregnancy Childbirth. 2023 Jun 20;23:459. doi: 10.1186/s12884-023-05764-6 (PMC10283212; doi:10.1186/s12884-023-05764-6)
Supplement: Supplementary file 1 — Supplementary Material 1 [file 12884_2023_5764_MOESM1_ESM.docx]

**Table S1: Distribution of couples’ blood group** **for RPL patients and controls.**

| **Wife's Blood Group (RPL patients and control) %** | **Husband's Blood Group in RPL patients and control group %** | | | | | | | |
| --- | --- | --- | --- | --- | --- | --- | --- | --- |
|  | **Patient BG (A)** | **Control BG (A)** | **Patient BG (B)** | **Control BG (B)** | **Patient BG (AB)** | **Control BG (AB)** | **Patient BG (O)** | **Control BG (O)** |
| **Patients' BG (A)** | 16 |  | 2 |  | 3 |  | 14 |  |
| **Control's BG (A)** |  | 17 |  | 3 |  | 4 |  | 10 |
| **Patients' BG (B)** | 6 |  | 7 |  | 1 |  | 8 |  |
| **Control's BG (B)** |  | 6 |  | 5 |  | 1 |  | 4 |
| **Patients' BG (AB)** | 2 |  | 1 |  | 0 |  | 3 |  |
| **Control's BG (AB)** |  | 4 |  | 2 |  | 1 |  | 2 |
| **Patients' BG (O)** | 10 |  | 8 |  | 3 |  | 14 |  |
| **Control's BG (O)** |  | 14 |  | 4 |  | 4 |  | 19 |

Data are expressed in percentages (%). BG: blood group. Blood group combinations that cause incompatibility are highlighted in dark gray while compatible combinations are highlighted in light gray.

**Table S2: Couples’ blood group** **interactions, and particularly ABO incompatibility.**

| **Couples’ blood group** **interactions** | **RPL Patients (%)** | **Controls (%)** | ***P* value** |
| --- | --- | --- | --- |
| **Wife (A) with Husband (B)** | 2 | 3 | 0.651 |
| **Wife (B) with Husband (A)** | 6 | 6 | 1.000 |
| **Wife (O) with Husband (A)** | 10 | 14 | 0.385 |
| **Wife (O) with Husband (B)** | 8 | 4 | 0.235 |
| **Wife (O) with Husband (AB)** | 3 | 4 | 0.701 |
| **Total Incompatibility** | 29 | 31 | 0.758 |

Data are expressed as percentages (%). Statistical analysis was done using Chi-square.
